# Integrating functional genomics data using maximum likelihood based simultaneous component analysis

**DOI:** 10.1186/1471-2105-10-340

**Published:** 2009-10-16

**Authors:** Robert A van den Berg, Iven Van Mechelen, Tom F Wilderjans, Katrijn Van Deun, Henk AL Kiers, Age K Smilde

**Affiliations:** 1 SymBioSys, Katholieke Universiteit Leuven, Leuven, Belgium; 2 Heymans Institute, University of Groningen, Groningen, The Netherlands; 3 Biosystems data analysis, Swammerdam Institute for Life Sciences, Universiteit van Amsterdam, Amsterdam, The Netherlands

## Abstract

**Background:**

In contemporary biology, complex biological processes are increasingly studied by collecting and analyzing measurements of the same entities that are collected with different analytical platforms. Such data comprise a number of data blocks that are coupled via a common mode. The goal of collecting this type of data is to discover biological mechanisms that underlie the behavior of the variables in the different data blocks. The simultaneous component analysis (SCA) family of data analysis methods is suited for this task. However, a SCA may be hampered by the data blocks being subjected to different amounts of measurement error, or noise. To unveil the true mechanisms underlying the data, it could be fruitful to take noise heterogeneity into consideration in the data analysis. Maximum likelihood based SCA (MxLSCA-P) was developed for this purpose. In a previous simulation study it outperformed normal SCA-P. This previous study, however, did not mimic in many respects typical functional genomics data sets, such as, data blocks coupled via the experimental mode, more variables than experimental units, and medium to high correlations between variables. Here, we present a new simulation study in which the usefulness of MxLSCA-P compared to ordinary SCA-P is evaluated within a typical functional genomics setting. Subsequently, the performance of the two methods is evaluated by analysis of a real life *Escherichia coli *metabolomics data set.

**Results:**

In the simulation study, MxLSCA-P outperforms SCA-P in terms of recovery of the true underlying scores of the common mode and of the true values underlying the data entries. MxLSCA-P further performed especially better when the simulated data blocks were subject to different noise levels. In the analysis of an *E. coli *metabolomics data set, MxLSCA-P provided a slightly better and more consistent interpretation.

**Conclusion:**

MxLSCA-P is a promising addition to the SCA family. The analysis of coupled functional genomics data blocks could benefit from its ability to take different noise levels per data block into consideration and improve the recovery of the true patterns underlying the data. Moreover, the maximum likelihood based approach underlying MxLSCA-P could be extended to custom-made solutions to specific problems encountered.

## Background

In contemporary biology, it becomes more widespread to study complex biological processes by collecting and analyzing measurements on the same entities from different sources, such as transcriptomics, metabolomics, ChIP-chip, or proteomics. The data originating from such measurements can often be organized in matrices pertaining to experimental units (e.g., tissues or culture samples) and variables (e.g., genes or metabolites) that were measured on these experimental units. The experimental units, also referred to as objects, constitute the experimental mode of the data, and the measured biochemical compounds the variable mode. We will denote such matrices consisting of measurements originating from different sources by data blocks.

Data blocks with information on the same entities stemming from different sources share one of the data modes; as such we will further denote them by the term 'coupled data'. For instance, Ishii and coworkers [1] simultaneously collected metabolomics, transcriptomics, and proteomics measurements from *Escherichia coli *chemostat cultures with different mutants and environmental conditions. This yields measurements coupled via the experimental mode. Other examples of publications involving this type of data are [2,3]. As an alternative, data blocks can be coupled via the variable mode. This occurs, for instance, in experiments in which transcriptomics measurements are coupled with ChIP-chip measurements [4], or even with ChIP-chip and motif data [5].

Often, the purpose of collecting coupled data will be to discover biological mechanisms that underlie the behavior of the variables in the different data blocks. For example, when the measurements originate from experiments in which metabolomics and transcriptomics analyses were conducted, the researcher could be interested in identifying regulatory mechanisms that coordinate a joint response on metabolome and transcriptome level.

To arrive at a comprehensive synthesis of the information on biological mechanisms underlying coupled data blocks, the data blocks have to be analyzed simultaneously. For such a synthesis, the family of simultaneous component analysis (SCA) methods is a natural choice. SCA methods search for important patterns in the data blocks and reveal the contributions of the variables and the experimental units to these patterns, similar to principal component analysis (PCA). The identified patterns can subsequently aid the discovery of the regulatory mechanisms underlying the data.

However, a simultaneous analysis of multiple data blocks may be hampered by the data blocks being heterogeneous in a number of respects. For instance, measurements originating from different functional genomics platforms can be subject to different amounts measurement error, or noise related to the accuracy of the platforms in question.

The noise present in the different data blocks can obscure the data patterns. Therefore, it can become more difficult to extract information regarding these patterns. For this reason, it could be fruitful to take data block noise into consideration in the data analysis. In particular, when data blocks are subject to different amounts of noise, it seems desirable to treat the data block with more noise with more caution.

Yet, the different noise levels should be known to be able to take these into consideration. Often however, it is unknown how much noise is present in each data block. If this were the case, a method is needed that also estimates the noise in each data block. Such a method was proposed recently in the psychometrics field: MxLSCA-P, a maximum likelihood based SCA method (Wilderjans, T.F., Ceulemans, E., Van Mechelen, I., van den Berg, R.A.: Simultaneous analysis of coupled data matrices subject to different amounts of noise, submitted). MxLSCA-P explicitly estimates the noise levels per data block and integrates these estimations in the overall analysis. In a simulation study, MxLSCA-P outperformed standard SCA-P [6] when recovering the underlying structure of simulated data blocks that were subject to different noise levels.

One may wish to translate the results of the simulation study mentioned above to the analysis of coupled functional genomics data. There are, however, two obstacles that prevent a direct translation. First, the data blocks simulated in the previous study were coupled via the variable mode, while functional genomics measurements often pertain to measurements coupled via the experimental mode [1-3]. Different coupling leads to a rather different kind of analysis, in particular with regard to the type of preprocessing that is linked to different SCA methods [7-10]. It is therefore not self-evident that the previous results hold for data blocks coupled via the experimental mode. Second, the simulation study did not consider data aspects that are typical for functional genomics, such as, having more variables than objects, and moderate to high correlations between variables (e.g., between two co-regulated genes) as the simulation was based on randomly generated components.

In this paper we will present a new simulation study to overcome these obstacles and to ascertain the relevance of MxLSCA-P for the analysis of functional genomics data coupled via the experimental mode. For this purpose we will determine the performance of MxLSCA-P in a context in which (i) the experimental mode is shared; and (ii) the correlations between variables are realistic in that they mimic the correlations observed in a real life microbial metabolomics data set consisting of two coupled GC/MS (gas chromatography combined with mass spectrometry) and LC/MS (liquid chromatography combined with mass spectrometry) data blocks. In addition, we will also apply standard SCA-P and MxLSCA-P to the real life metabolomics data set itself. Before presenting the results of the analysis of simulated and real-life data sets, we will now first explain SCA-P and MxLSCA-P. Subsequently, we will outline the problem and setup of our new simulation study.

### Simultaneous component analysis

#### Notation

In this paper matrices and vectors will be indicated by bold uppercase and lowercase characters as in Kiers [11]. Elements will further be denoted by lowercase running indices that range from 1 to the corresponding uppercase characters. For instance, the number of objects in a data block will be indexed by *i*, running from 1 to *I*.

#### General SCA decomposition

The family of SCA methods [10] comprises a wide range of component methods that share two characteristics. First, they reduce the dimensionality of the data blocks by decomposing the data blocks in components, and second they do so while minimizing the loss of information. The SCA methods distinguish themselves from other components methods [10] by (i) *simultaneously *decomposing coupled data blocks with the different data blocks taking exchangeable roles, and (ii) allowing for block-specific weighting of data blocks to capture particular aspects of the data blocks more adequately.

In general, given a set of *K *data blocks ***X***_*k *_that share an object mode with *I *objects and *J*_*k *_variables, and a set of prespecified block-specific weights *w*_*k*_, a SCA decomposition reads as follows:

(1)

with **T**(*I *× *R*) denoting a score matrix for *R *components shared by all *K *data blocks, **P**_*k*_(*J*_*k *_× *R*) the accompanying block-specific loadings, and **E**_*k*_(*I *× *J*_*k*_) a residual matrix.

This decomposition of data blocks that share the object mode will be the reference decomposition in this paper. For other situations in which the data blocks share a variable mode, the SCA decomposition is given by:

(2)

with **T**_*k*_(*I_*k *_× R*) denoting a block-specific score matrix for *R *components, **P**(*J × R*) the loadings shared by all data blocks, and **E**_*k*_(*I*_*k *_× *J*) a residual matrix.

#### Model estimation

For the estimation of **T **and **P**_*k *_the following objective function is minimized:

(3)

The optimal matrices **T **and **P**_*k *_that minimize (3) can be estimated on the basis of the following identity:

(4)

Where **X**_*c *_= [*w*_1_**X**_1_...*w*_*k*_**X**_*k*_...*w*_*K*_**X**_*K*_] with size  is the concatenation of all *w*_*k*_**X**_*k*_, and  with size  is the concatenation of all **P**_*k*_; the estimates then can be obtained by means of a singular value decomposition (SVD) [10]. For identification purposes, the components can be constrained to have a principal axis orientation and **T **or **P**_*c *_to be orthonormal.

The SVD of **X**_*c *_reads as follows:

(5)

If **T **is chosen to be columnwise orthonormal, **T **can be obtained by choosing the *R *left singular vectors associated with the *R *largest singular values in **S**. The loadings **P**_*c *_are then obtained by multiplication of the *R *right singular vectors with the *R *associated largest singular values:

(6)

where the subscript '*R*' indicates the *R *largest singular values and accompanying singular vectors. In case **P**_*c *_is chosen to be orthonormal, **P**_*c *_is put equal to **V**_*R *_and **T **to **U**_*R*_**S**_*R*_.

#### SCA with equal block weights

SCA with equal block weights (*w*_1 _= ... = *w*_*K *_= *w *> 0) was proposed in the psychometrics literature as SCA-P [6] and in the chemometrics literature as SUM-PCA [12]. Both methods fit the general SCA decomposition as methods in which equal weights are applied to the different data blocks. In the remainder of this paper we will refer to this method as SCA-P.

Choosing equal block weights implies that all the data entries in the different data blocks are considered equally important and that no further block-specific adjustments are made to increase or decrease their relative influence. This approach was coined a 'one entry, one vote' approach [13]. The objective function of this method is:

(7)

#### MxLSCA-P

MxLSCA-P (Wilderjans, et al.: submitted) is a stochastic extension of the generic SCA method (1). Unlike SCA-P, it assumes that the residuals in **E**_*k *_follow a normal distribution with a mean of zero and an unknown block-specific variance:

(8)

The minus loglikelihood function for the MxLSCA-P method is (Wilderjans, et al.: submitted):

(9)

in which *c *denotes a constant term that does not influence the minimization of the minus loglikelihood function. (This equation generalizes the equivalent equation in (Wilderjans, et al.: submitted) that pertained to the two block case. We minimize the minus loglikelihood in line with the optimizations discussed previously.) The improved performance of MxLSCA-P in the previous simulation study (Wilderjans, et al.: submitted) can be understood from the different model assumptions made. SCA-P implicitly assumes that noise across the different data blocks is identically distributed, i.e., it maximizes the likelihood function based on the assumption that the noise is distributed identically in the different data blocks. When this assumption is violated and the noise is distributed differently, the SCA-P model becomes misspecified, unlike MxLSCA-P that specifically allows for those differences.

The objective function of MxLSCA-P (9) differs from the general objective function for SCA methods (3) by the introduction of block-specific noise parameters *σ*_*k*_. These noise parameters act as a weight to the data blocks and in a new term '*IJ*_*k *_log *σ*_*k*_'. Unlike in the general SCA decomposition, in MxLSCA-P the block weights are to be estimated as an integrated part of the analysis.

The parameters of MxLSCA-P (*σ*_*k*_, **T**, and **P**_*k*_) cannot be estimated directly via an SVD. Therefore an alternating least squares (ALS) algorithm [14,15] was developed (Wilderjans, et al.: submitted).

In an ALS algorithm, the parameters to be estimated are split into subsets that are alternatingly re-estimated conditionally on each other. In particular, the following procedure is followed:

1. The algorithm is initiated by choosing values for *σ*_*k*_. These starting values for *σ*_*k *_can be determined randomly or rationally (e.g., based on a SCA-P). It is advised to use multiple different starting values to avoid getting stuck in local minima.

2. The scores **T **and loadings **P**_*k *_are estimated conditional on the values of *σ*_*k *_via an SVD. This SVD optimizes the following part of the objective function: .

3. New estimations  of *σ*_*k *_are calculated conditional on the previous estimations of  and :

(10)

4. The current value of the objective function (9) is calculated.

The second, third, and fourth step are repeated until a convergence criterion is met (e.g., changes in the objective function below a prespecified threshold).

### Problem and setup of the simulation study

A simulation study was set up to assess the performance of the SCA-P and MxLSCA-P methods for the analysis of functional genomics data blocks coupled via the experimental mode. The performance of the methods was evaluated in terms of their ability to recover the true structures (**T**^*m*^, , , , and ) underlying two simulated data blocks subject to different simulation settings. To improve the realism of the simulations, the data blocks were simulated using the correlation structure of the variables as observed in a real life GC/MS and LC/MS microbial metabolomics data set (see Methods section).

Furthermore, different data characteristics that could influence the analysis of coupled functional genomics data blocks were varied. In particular, the following characteristics were included as design factors (see Methods section for detailed information):

• Noise level of the data blocks. Noise can hamper the recovery of the true data structures, especially if the noise levels of different coupled functional genomics data blocks would differ. In the simulation study noise was manipulated via two factors: (i) the noise ratio between the two data blocks (factor Noise Ratio), and (ii) the total amount of noise on the data blocks (factor Noise Total).

• Different numbers of variables per data block. In functional genomics research, different data blocks can considerably differ in the number of variables (e.g., metabolomics and transcriptomics data sets can consist of hundreds and thousands of variables, respectively). Moreover, the number of variables is generally larger than the number of objects which induces collinearity in the data [16,17]. A SCA can be influenced by these factors in two ways. First, when the difference between the number of variables in different data blocks is large, the larger data block could dominate the analysis. Second, induced collinearity may hamper a correct estimation of the loadings.

In this simulation study a small and a large data block were simulated with different numbers of variables per data block (factor Number of variables). The total number of variables was always larger than the number of objects such that collinearity was always present. The large data block used the correlation structure observed in the GC/MS data set and the small data block the correlation structure of the LC/MS data set.

• Relative importance of the data blocks. The variation present in one data block, and thus its importance, can differ from other data blocks. This could influence the recovery of the data structures, as data blocks with high variation can dominate other data blocks. The variation present in the data blocks is in an SVD expressed by the singular values. Here, the relative importance of a data block was manipulated by these singular values (factor Singular value).

In addition to these factors, a factor Methods was included in the experimental design, with SCA and MxLSCA-P as its two different levels. Recovery performance and the impact on it of the factors manipulated in the simulation study were analyzed by means of an analysis of variance (ANOVA).

## Results

### Performance of the SCA methods on simulated data

The recovery by the two SCA methods of the true data structures as measured by a Fisher-Z transformed modified RV coefficient [18] (RV-Z) was generally good. Recovery performance appeared to depend both on the specific structural aspect looked at, and on data characteristics as manipulated in the simulation study (Table 1). Below we will discuss the different data characteristics and their influence on the recovery of the true structural aspects.

**Table 1:** Excerpt from the ANOVA tables of the analysis of the recovery of the true structures underlying the simulated data.

**True structure**	**Factor**	**df**	**F**	*ω*^2^
**T**^*m*^	Noise Total	2	139 855	.44
	Method	1	106 207	.17
	Noise Ratio * Noise Total	4	26 255	.17
	Method * Noise Ratio	2	23 912	.075
	Method * Noise Total	2	22 988	.072

	Noise Total	2	370 120	.36
	Noise Ratio	2	361 152	.36
	Noise Ratio * Noise Total	4	141 444	.28

	Noise Total	2	112 233	.37
	Noise Ratio	2	107 744	.35
	Noise Ratio * Noise Total	4	42 273	.28

	Noise Total	2	39 177	.41
	Noise Ratio * Noise Total	4	10 644	.22
	Noise Ratio	2	9 239	.096
	Method	1	16 792	.088
	Method * Noise Ratio	2	6 599	.069

	Noise Total	2	21 643	.39
	Noise Ratio * Noise Total	4	7 018	.26
	Method * Noise Ratio	2	4 622	.084
	Method	1	8 369	.076
	Noise Ratio	2	4 169	.076

F denotes the value of the F statistic, df the degrees of freedom, and *ω*^2 ^the effect size. Only the most important factors in terms of *ω*^2 ^(*ω*^2 ^≥ .050) are reported (all were significant *p *< .0001).

**Figure 1:**
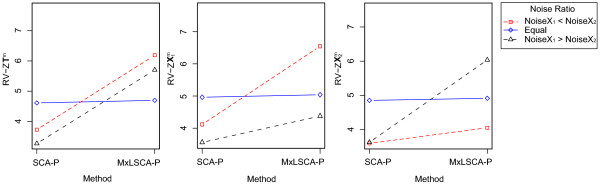
**Mean recovery of T^*m*^, , and  for all combinations of the levels of 'Method' and 'Noise Ratio'**. The recoveries of the different true structures **T**^*m*^,  and  are given from left to right, respectively. The RV-Z is indicated on the y-axis. The two levels of 'Method' are indicated on the x-axis. The different lines indicate the different levels of the factor Noise Ratio (red, dashed, square = Noise**X**_1 _< Noise**X**_2_; solid blue, diamond = Equal; black, dashed, triangle = Noise**X**_1 _> Noise**X**_2_).

Most importantly for the purpose of this research, the main effect of factor 'Method' and its interaction with 'Noise Ratio' appeared to be sizeable on the level of the recovery of the true scores (**T**^*m*^) as well as of the true data block entries (, and ). In particular, MxLSCA-P performed on average significantly better than SCA-P (Table 2). Moreover, as appears from Figure 1, in the case of the recovery of **T**^*m*^,  and , MxLSCA-P especially outperforms SCA-P when the noise levels for the data blocks differ. For the recovery of **T**^*m *^(Figure 1, left panel), recovery was best when the largest data block, , was the least noisy. The recovery of a particular data block was further best (in absolute as well as relative sense) when that data block was subject to the least amount of noise (Figure 1, center panel: , right panel: ). Furthermore, the interaction between 'Method' and 'Noise Total' was also sizable or the recovery **T**^*m*^. This interaction showed that the benefit of MxLSCA-P is largest when the total noise level is low and the benefit becomes smaller for higher total noise levels. The advantage of MxLSCA-P over SCA-P for recovering the true underlying structures in the presence of different noise levels did not carry over to the recovery of the block-specific loadings  (Table 2). One might conjecture that this result is due to differences in the number of implicit constraints on the different constituents of the MxLSCA-P decomposition. The scores of the SCA decomposition are constrained to be identical for all data blocks; as a result, these scores may be prevented to be misguided by the data. The loadings, however, are not subject to such restriction, and, as a result have more freedom to deviate from the true model structure.

**Table 2:** Mean recoveries (RV-Z) for the levels of the design factor Method for the recovery of the true structures T^*m*^, , , , and .

**True structure**	**Method**	**Recovery (RV-Z)**	**SE**
**T**^*m*^	SCA-P	3.9	.0036
	MxLSCA-P	5.5	.0036
	SCA-P	4.2	.0075
	MxLSCA-P	5.3	.0075
	SCA-P	4.0	.0075
	MxLSCA-P	5.0	.0075
	SCA-P	4.3	.0022
	MxLSCA-P	4.3	.0022
	SCA-P	4.3	.0039
	MxLSCA-P	4.4	.0039

SE denotes the standard error. RV-Z values of 3.8 and 5.0 correspond to modified RV coefficients of 0.9990 and 0.9999, respectively. The differences between SCA-P and MxLSCA-P are significant for T*m*, , and  (p < .05).

**Figure 2:**
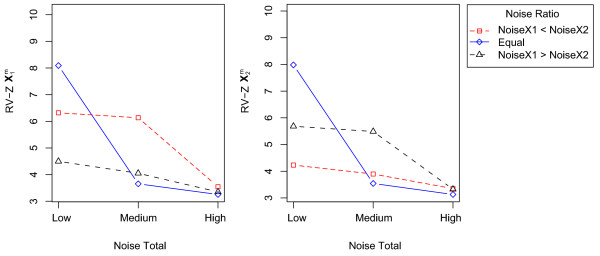
**Mean recovery of  (left panel) and  (right panel) for all combinations of the levels of 'Noise Total' and 'Noise Ratio'**. The RV-Z is indicated on the y-axis. The three levels of 'Noise Total' are indicated on the x-axis. The different lines indicate the levels of the factor Noise Ratio (red, dashed, square = Noise**X**_1 _< Noise**X**_2_; solid blue, diamond = Equal; black, dashed, triangle = Noise**X**_1 _> Noise**X**_2_). The RV-Z values were averaged over the other factors, e.g., the factor Method.

A sizeable main effect of 'Noise Total' was found for the recovery of all true structural aspects. This effect is obvious with more noise leading to a poorer recovery. Furthermore, for the recovery of all block-specific structural aspects (i.e., the true loadings , , and the true data block entries of  and ), the main effect of 'Noise Ratio' was important as well, with the true structures being recovered better when the corresponding data block was less noisy. Furthermore, the interaction between 'Noise Total' and 'Noise Ratio' was substantial for the recovery of all data structures. This interaction was plotted in Figure 2 for the cases of the recovery  and  (for the block-specific loadings the pattern was similar). From Figure 2 it becomes clear that the effect of 'Noise Ratio' (i.e., better recovery when a particular block is relatively less noisy than the other as compared to a situation with a reverse noise ratio) shows up only in case of low to medium noise levels. In addition, a very good recovery is observed in case of the combination of a low total noise level and a Noise Ratio of 1; the latter is due to the fact that this particular combination implies a very low total noise level for the whole of the two data blocks (10^-3^%). For the recovery of the common scores, the interaction between 'Noise Total' and 'Noise Ratio' took a slightly different shape: Now the two conditions of 'Noise Ratio' that implied different noise levels for the two data blocks resulted in a better recovery in case of low and medium 'Total noise' levels.

### Analysis of real life microbial metabolomics data

To obtain an as complete as possible overview of the changes of the concentrations of metabolites in microbial metabolomics, multiple analytical platforms are required [19]. In this paper, *E. coli *metabolomics data consisting of metabolite concentrations that were obtained using GC/MS and LC/MS [20] were used. The data set consisted of 28 samples of batch fermentations with varying experimental conditions (e.g., low oxygen, succinate or D-glucose as sole carbon source, wild type or phenylalanine overproducing strain) taken at different time points. In general, different analytical platforms can perform differently with regard to reproducibility. Therefore, the analysis could potentially benefit from an MxLSCA-P approach that takes noise heterogeneity into account.

**Figure 3:**
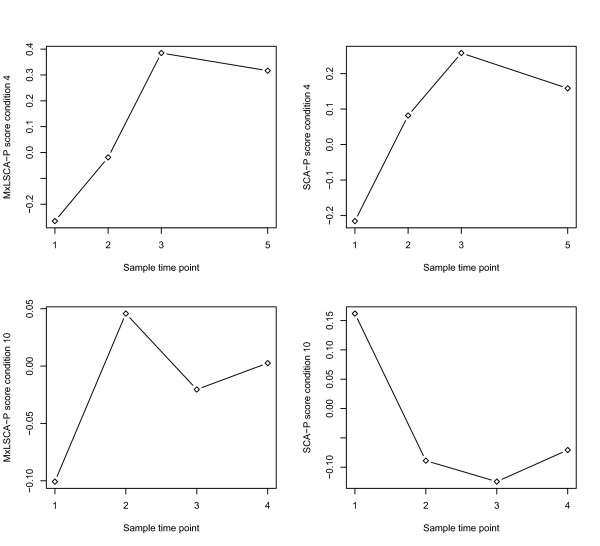
**Scores on the third component of MxLSCA-P and SCA-P**. Scores on the third component of MxLSCA-P (left) and SCA-P (right) for experimental conditions 4 and 10 (from top to bottom the first and second row of panels, respectively). On the x-axis, the different time points of sampling are presented ranging from 'early' (1) to 'late' (3, 4, and 5). The y-axis indicates the score value in arbitrary units.

We subjected the data under study to MxLSCA-P and SCA-P analyses with three components. The three components were selected based on the scree plots of component analyses of the individual data blocks. Subsequently, the MxLSCA-P and SCA-P score plots were compared. The first two components appeared to be very similar: On the first component the samples obtained from succinate grown cells differed strongly from the other samples; the second component showed a separation between samples obtained under low oxygen conditions and samples obtained at late time points of both succinate grown cells and wild type cells. However, differences between the two methods became apparent for the third component. In particular, the scores on the third MxLSCA-P component for those conditions for which multiple time points were sampled as a function of time were plotted. For all these plots, profiles resembling typical batch fermentation growth curves were found. In such a growth curve, the cells first grow fast as a sufficient amount of nutrients is available; next, when nutrients become depleted, growth is halted and the curve starts to decline. A typical example of such a profile in the MxLSCA-P scores is plotted in the upper left corner of Figure 3. For SCA-P, such typical profiles were also found for five experimental conditions (see e.g., Figure 3, upper right plot), but for two experimental conditions the patterns differed (see e.g., Figure 3, lower right plot). (Note that the profile in the lower right plot cannot simply be reflected to match the typical batch fermentation profile, as reflections of SCA scores and loadings can only be performed on the entire score or loading vector and not on a subset of it.)

The pattern of the block-specific loadings further nicely complemented the pattern of the scores. In particular, inspection of the loadings on the third component for the LC/MS data block revealed high contributions for cell wall precursors for peptidoglycan biosynthesis [21,22] (like UDP-N-AAGDAA and UDP-N-AAGD) and nucleotides (such as, UDP, UTP, CMP, CDP, and CTP) that are involved in a wide range of cellular processes, among which cell wall biosynthesis [22]. Cell wall biosynthesis can be linked to the growth phases in a batch fermentation, as metabolites involved in it are likely to fluctuate depending on these growth phases. For instance, during exponential growth, cell wall intermediates are required for growth and cell division, whereas during the stationary growth phase the demand for these intermediates is expected to drop.

The MxLSCA-P block-specific loadings for the third component pertaining to the GC/MS data block revealed consistently large contributions for uncharacterized disaccharides; for the corresponding SCA-P loadings this was less clearly the case. Within the context of this study, there are two likely roles for disaccharides in *E. coli*, which both could relate to variation in metabolite concentrations during the different phases of a batch fermentation: (i) In cell wall biosynthesis, the different parts of the cell wall have polysaccharides as a major constituent, for instance, in peptidoglycan [21,22] and in lipopolysaccharides [22,23]. (ii) Disaccharides could play a role in the internal storage of excess carbon source, during conditions under which another nutrient excluding carbon source is limiting.

Summarizing, in this case study MxLSCA-P seemed better able to extract biologically relevant information. MxLSCA-P provided a more consistent link to the growth phases of the batch fermentations, both through the common scores and through the LC/MC data block loadings. Also, the disaccharides involved in the MxLSCA-P loadings for the GC/MS block are likely to link up with cellular processes related to the different batch fermentation growth phases.

## Discussion

MxLSCA-P was proposed to model coupled data blocks with heterogeneous noise levels. In a previous simulation study, MxLSCA-P was shown to outperform SCA-P in recovering the true structure underlying the data that did not consider typical problems encountered in functional genomics studies. In the study presented in this manuscript the previous study was extended to address these problems typical for functional genomics: (i) the data were coupled via the experimental mode, (ii) the simulations were based on correlation structures observed in real life data sets, (iii) collinearity was induced by ensuring the data had more variables than objects. Our results showed that MxLSCA-P also outperforms SCA-P in simulated data that mimic functional genomics data more closely. In particular, MxLSCA-P was better able to recover the true scores (**T**^*m*^) and true data blocks ( and ) especially when the relative noise levels differed across data blocks. Furthermore, MxLSCA-P provided a more consistent and biologically more meaningful interpretation of the analysis of the *E. coli *metabolomics case study. Therefore MxLSCA-P seems to be the preferred choice over SCA-P for the kind of data we have studied, but probably for other kinds of data as well.

In SCA-P, the data blocks are given equal *a priori *block weights as there is no *a priori *reason to treat the data blocks differently. MxLSCA-P is an extension of SCA-P in which, as an integrated part of the analysis, the equal *a priori *block weights are combined with data-driven *a posteriori *weights that reflect the noise levels of the different data blocks such as to de-emphasize the most noisy data blocks. Within the family of SCA methods, other methods exist that *a priori *weigh the data blocks differently to ensure that each block makes a "fair" contribution to the analysis. Such a weighting can be based on different conceptions of fairness [10], for instance, to ensure that each data block has the same amount of variation [12], or that data blocks with more redundant information are downweighted [24]. (The latter conception is the basis of multiple factor analysis, which was recently applied for the analysis of coupled functional genomics data blocks by de Tayrac and coworkers [25]). Those *a priori *weights to ensure a fair block weighting, however, do not take into account differences in measurement error, or noise levels. Indeed, analogous to SCA-P, in other SCA methods it is implicitly assumed that the data blocks have equally and independently normal distributed noise levels. Therefore, these other SCA methods, too, could potentially benefit from block-specific noise estimations on the basis of maximum likelihood extensions as discussed in the present paper. Following such an approach, the *a priori *fairness correction could be blended with block-specific noise estimations.

SCA-P assumes that the noise levels are equal for all data blocks. Often, this assumption does not match with situations encountered in practice. MxLSCA-P addresses this problem by allowing for different noise levels per data block, and by only requiring that the noise levels within each data block are equally and independently normal distributed. Yet, it is possible that noise levels also vary within a data block. For example, in addition to the fact that different measurement platforms can have different levels of reproducibility on average, within a measurement platform some variables could be measured more or less reliably than others (e.g., because of their chemical properties). This example illustrates that MxLSCA-P could benefit from allowing more complex 'within data block' error variance structures. Such complex variance structures could be incorporated following, for instance, a generalized least squares approach [26,27].

Research within the functional genomics field is not only limited to static experiments, experiments in which samples are obtained in time are also often conducted (e.g., [28,29]). To discover time-related effects in the data, MxLSCA-P could be extended using functional data analysis approaches [30].

Sometimes, the data sets collected in functional genomics studies are incomplete and contain missing data entries, for instance, due to experimental complications. The MxLSCA-P method could be extended to handle data sets containing missing values. For this, strategies like criss-cross regression [31,32] could be adapted.

## Conclusion

MxLSCA-P is a promising addition to the SCA family. Its ability to take different noise levels per data block into consideration and improve the recovery of the true patterns underlying the data could be beneficial for the analysis of coupled data blocks originating from different functional genomics sources. Moreover, the maximum likelihood based approach to SCA offers room for further extensions to allow for custom-made solutions to specific problems encountered in functional genomics research.

## Methods

### Metabolomics data

The metabolomics data set consisted of *E. coli *metabolomes (*E. coli *NST 74, a phenylalanine overproducing strain, and *E. coli *W3110, the wild-type strain). The *E. coli *strains were grown under different experimental conditions as described elsewhere [20]. The samples were analyzed by GC/MS and [33] and LC/MS [34]. The GC/MS and LC/MS samples were measured in duplicate. The final data blocks were manually cleaned up, removing spurious and double entries. After averaging of the duplicate measurements the data consisted of 28 experiments, 131 metabolites measured by GC/MS, and 44 metabolites measured by LC/MS. The metabolite data were autoscaled before analysis with SCA-P and MxLSCA-P. After autoscaling, each variable had mean zero and standard deviation one.

### Simulation study

#### Experimental design

A full factorial design was developed for the simulation study. Each cell of the experimental design was independently repeated 20 times. The design consisted of the following factors:

• The first factor is 'Method' with the two levels referring to the two different methods, SCA-P and MxLSCA-P.

• The second factor is 'Noise**X**_1_'. This factor determines the amount of noise on , the first simulated data block (see (11)). The levels of this factor are 10^-3^, 6.67, and 13.33% of noise variation of the total variation of  block. The specific percentages were chosen to simplify the conversion of data block noise levels into the factor 'Noise Total' (see below).

• The third factor is 'Noise**X**_2_'. This factor determines the amount of noise on  and has the same levels as the factor 'Noise**X**_1_' now pertaining to **X**_2_.

• The fourth factor is 'Number of variables' per **X **block. The first and second number indicates the number of variables of  and , respectively. The levels are '100 - 10', '70 - 20', and '40 - 30'.

• The fifth factor is the factor 'Singular value' and its three levels are '4, 2 & 2, 1'; '2, 1 & 2, 1'; and '2, 1 & 4, 2'. The first two values become the singular values of , the true **X**_1 _data block, and the second two become those of . Thus, for the first level of this factor,  receives singular values 4 and 2, and  2 and 1. Note that these singular values are scaled to correct for the number of variables in each block before they become the final singular values of the **X **block (see section Data generation).

To improve the interpretation of the effect of different noise levels on the recovery of the true underlying data structures, the noise factors of the experimental design were converted into a 'noise ratio between data blocks (Noise Ratio)' and a 'sum of the noise levels (Noise Total)' factor. These factors were not part of the simulation, but were used instead of the factors 'Noise**X**_1_' and 'Noise**X**_2_' as independent variables in the ANOVA:

• The Noise Ratio between data blocks factor consisted of three levels:

Noise**X**_1 _< Noise**X**_2_, Equal, and Noise**X**_1 _> Noise**X**_2_.

• The Noise Total factor consisted of 'Low', 'Medium', and 'High' noise levels over all the blocks. In this study, 'Medium' was equal to Noise**X**_1 _+ Noise**X**_2 _= 13.33. The sum of the noise levels smaller than 13.33 was 'Low', and larger was 'High'.

These converted factors remained orthogonal to the other design factors and to each other.

#### Data generation

Generation of the data blocks under the experimental design relied on Equation (11)

(11)

where  and  are generated under the design factors, and the matrix  refers to the *k*^th ^simulated data block. The true model parameters are indicated by 'm'. For completeness, the true data block  is given by . The simulation study was performed in Matlab R2008a (the Mathworks).

The true loading matrices  and  were generated based on the correlation matrices of the real life metabolomics data blocks. The data block obtained by GC/MS consisted of more variables than the LC/MS data block. Therefore, the GC/MS data block was used in the generation of the loadings for the largest data block in this simulation, , and the LC/MS data block was used for the generation of . The following procedure was followed in each simulation for the generation of the loadings:

• Randomly select *J*_*k *_variables from . The label 'real' indicates that these variables pertain to the real life measurements. The number of variables *J*_*k *_was given by the relevant design factor. Note that care was taken that *J*_*k *_is sufficiently smaller than  to ensure the subset of selected variables was sufficiently different in each simulation.

• Calculate the correlation matrix (*J*_*k *_× *J*_*k*_)

• Extract two normalized singular vectors belonging to the two largest singular values of . These two vectors form (*J*_*k *_× 2).

• Obtain the diagonal matrix  (2 × 2) based on the factor 'Singular value'. Scale  by multiplying  by  to correct for differences in block size.

• Obtain the true loading matrix  (*J*_*k *_× 2) by multiplication of  with : .

For each simulation, the true score matrix **T**^*m *^(20 × 2) were obtained from the left singular vectors of a centered matrix of which the elements were independently drawn from a standard normal distribution. The elements of the noise matrix  (20 × *J*_*k*_) were each simulation obtained by independently drawing values from *N*(0, ). The variance parameter  was set such that the expected variation of  was a certain percentage of the total variation. This percentage was given by the design factors Noise**X**_1 _and Noise**X**_2 _for respectively the largest and smallest data block.

#### Recovery of the true data structures

As performance measure for the different methods, the recovery of the true component matrices **T**^*m *^and  and the true data blocks  from the simulated data block  by the different SCA methods was determined. The closer the estimation of the components resembled the true component matrices, the better a method performs. The recovery of the data structures was measured by the modified RV coefficient [18], a matrix correlation measure, as a goodness of recovery measure. The range of modified RV coefficient is between -1 and 1 and '1' means perfect recovery. The modified RV coefficient is insensitive to orthogonal rotations, therefore we expect values close to 1. The modified RV coefficients were transformed using the Fisher-Z transformation to allow for values on the entire real line instead of between -1 and 1, thus a larger number indicates a better recovery. The transformed values are referred to as RV-Z. The recovery of the true data blocks  was also analyzed by the sum of squared differences per data block. This different recovery measure did not change the conclusions of this paper. Therefore, the RV-Z measure was used as a recovery measure for all data structures. The recovery measures obtained from the simulation study were analyzed by ANOVA using the GLM procedure of the software package SAS 9.2 (SAS). All factors were considered fixed for the ANOVA.

## Authors' contributions

RVDB performed the simulations, the analysis of the metabolomics data, and the writing of the manuscript. IVM recognized the usability of MxLSCA-P for the analysis of functional genomics data and aided the interpretation of the results and the writing of manuscript. TFW provided useful suggestions for the setup of the simulation study and the interpretation of the results. KVD provided useful suggestions for the setup of the simulation study, the ANOVA, the interpretation of the results, and the writing of the manuscript. HALK and AKS provided useful suggestions for the interpretation of the results. All authors read and approved the final manuscript.
